# Importance of Nourishment in Fever

**Published:** 1855-05

**Authors:** 


### THE GREAT IMPORTANCE OF NOURISHMENT IN FEVER.

BY DR. STOKES.

I wish also strongly to impress on you the great importance of the use of other forms of nourishment in this disease; for we must not only keep up the nervous energy of the system by wine, but we must support nature by food. There is no mistake more fatal in fever than the withholding of food. I was early taught the importance of the use of careful nourishment in fever by my friend and colleague, Dr. Graves. I remember once, Dr. Graves, when speaking of the use of nourishment in fever, made use of these words,—"If you are at a loss for an epitaph to be placed on my tomb, here is one for you,—*He fed fevers.*" I addition to the prejudices with which the inflammatory doctrine imbued so many minds, with respect to the use of food in fever, there was a new set of arguments raised against it, in consequence of the experiments of an American physician. I allude to the case observed by Dr. Beaumont, and so often quoted since. In this remarkable case, various medical substances and articles of food were introduced through an external fistula into the stomach, their effects being noted, as also the conditions of temperature, vascularity, &c. A set of results were subsequently published in connection with the action of the stomach upon food. One of the results stated to have been thus obtained was, that the existence of the state of fever altogether suspended the process of digestion. Here was a statement which had the appearance of being the result of strict observation. It influenced a number of young men; but did it influence those who had once been in charge of a fever hospital? Not at all; because those men knew very well that, no matter what Beaumont might say about the stomach not digesting when the patient had fever, in thousands of cases patients in fever digested remarkably well, required food, and derived benefit from it. In a large number of cases of typhus fever, the stomach has an excellent power of digestion; and, I believe, if we were bold enough, we would find that many articles of food usually forbidden to fever patients might have been given to them with safety. A curious incident was related to me which shows that the stomach in fever is capable of digesting even a rather coarse article of food. A lady who had been recently married was attacked with extremely severe petechial fever; she was covered with dark-colored maculæ, and the disease had run to about the twelfth or thirteenth day. She was attended by several eminent physicians. Her case was an extremely bad one, and her life was all but despaired of. She was violently delirious. Her husband had occasion to leave the house, on some business. At the period of the dinner-hour of the family, the servants were cooking a rump of beef and cabbage, and the odor of it filled the house. In her delirium she called for some of the beef and cabbage; she was then, you must understand, in severe fever, and covered with maculæ. Her sister, who was attending her, believing she was dying, thought it only right to indulge her, from the feeling that it was right to indulge the request of a dying person. She proceeded to the kitchen; and, as soon as the beef was boiled, cut a very large mess of beef and cabbage; this was brought up smoking hot to the lady's bed-side, when she devoured it with great avidity. Shortly afterwards her husband came in, and was told what had happened. He became terrified, and sent for physicians in every direction. Four or five assembled; time was pressing, and every one agreed that something should be done. At length the late Dr. Harvey, a practical physician of the very first class, arrived. He was laid hold of by the agonized husband, forced up-stairs, and his opinion earnestly requested. At that time the stomach-pump was not in fashion, but every one agreed that something decisive should be done, that an emetic should be given, or some extraordinary effort made to get this mess of beef and cabbage out of the lady's stomach. When Dr. Harvey went to the bed-side, he found the patient in a tranquil sleep. He turned round, and when anxiously appealed to what should be done, he said—"You had better wait till she awakens; let her sleep it out." She slept for four or five hours; awoke wonderfully better, and on the following day was out of danger. I

do not give this case to induce you to feed your patients with salt beef and cabbage in fever, but it is very important, as showing that in typhus fever with maculæ, the stomach is capable not only of digesting a coarse article of food as salt beef, but that even such food may have a good effect.—(*Clinical Lectures on Fever, No.* 5.)

---

### INDICATIONS FOR TREATMENT IN FEVER AFFORDED BY THE HEART.

BY DR. STOKES.

"I have sometimes observed that students were under a misapprehension about the doctrines which we have long held in this hospital with respect to the condition of the heart as a guide for the use of wine. They have come to the erroneous opinion that we are only to give wine where we find the want of the first sound of the heart, and that we are not to give wine where the heart is acting well. This is a mistaken view of the matter. What we have established as to the state of the heart in connection with the effect of stimulants, is simply this,—we have ascertained that the efficacy of stimulants is often directly as the debility of the heart. It has been also ascertained that the power of bearing stimulants, their effect upon the nervous system, their good effects on the general condition, are directly as the weakness of the heart.—We may lay down as a rule, that there are three conditions of the heart to be looked at by the practical man in the treatment of fever. In one, we have an excited heart—a violently excited heart all through the case ; and this heart may be excited and violent, although the symptoms be those of extreme adynamia, although the surface be cold, the breath cold, and the pulse so feeble that it cannot be discovered. Nay, the heart may act with great force for several days, and yet there be no pulse at the wrist. This is one case. In the next case we find exactly an opposite condition, in which the systolic force of the heart is diminished. This is shown by loss of impulse of the heart, by diminution of the first sound, and, in certain cases by extinction of the first sound of the heart while the second remains. This is a case which calls for wine, and in which you should give it: it is a case in which, in the vast majority of instances, wine will agree with the patient. There is a third set of cases in which the heart does not seem to be implicated at all in the course of the disease, in which, notwithstanding the existence of the most extraordinary group of symptoms affecting various organs, the heart, in the middle of the storm, seems to be in a state of calm and quiet. If we compare these three sets of cases with a view to prognosis, we may arrange them in this way. The case of excited heart all through, with feeble pulse and with adynama, is unquestionably the worst case. There is no worse symptom in fever than an excited heart. It is especially a bad symptom when, with that excitement, we find a feeble pulse. The next will be the case of sinking of the heart; and the most favorable case is that in which, as I said before, the heart seems to escape disease. But you are not to suppose, that because you have an excited heart you are not to give wine if the symptoms of the patient require it: and you are not to suppose that, because the heart is not affected at all, you are to withhold wine if the general symptoms of the patient require it. You are not to found your exhibition of wine or stimulants upon any one thing; you are to take the general state of the patient into consideration. What we have done is to discover an intelligible practical rule which will guide you in the use of wine in certain, I think in many, cases; but you are not to suppose that because this man has a clear first sound at his heart, therefore you are not to give wine. You are not to suppose that because the heart is safe you can do without wine."—*Clinical Lectures on Fever.—From Ranking.*

